# Understanding post-hospitalised patients’ experiences of long COVID – the PELCO study

**DOI:** 10.1177/13591053241272233

**Published:** 2024-08-22

**Authors:** Alice Milne, David Arnold, Andrew Moore

**Affiliations:** University of Bristol, UK

**Keywords:** COVID-19, existential crisis, identity, long COVID, patient advocates, post-COVID syndrome, post-hospitalisation experience, psychological support, qualitative interviews, uncertainty

## Abstract

Despite significant advances in long COVID research, many aspects of the condition remain unknown. There is a persisting need for further research to improve the management of long COVID symptoms. This study aimed to explore the experiences and psychological needs of patients who were previously hospitalised with COVID-19, and who subsequently developed long COVID symptoms. Twelve patients with long COVID were interviewed between October 2021 and June 2022. Transcripts were analysed thematically. An overarching theme of ‘*Existential Crisis*’ was developed, incorporating three interconnecting sub-themes: ‘*Facing Psychological Threat*’, ‘*Seeking Legitimisation*’ and ‘*Forging a Path Through Uncertainty*’. Findings suggest that the psychological impact of emergency hospitalisation for COVID-19 can be severe, particularly for those with ongoing long COVID symptoms, and that early psychological intervention should be available. Our findings also suggest the importance of further planning for future pandemics to ensure the presence of patient advocates during hospitalisation at points of critical decision-making.

## Introduction

In November 2020, the UK’s National Health Service first announced initial provision of specialist long COVID clinics in recognition of the growing need to address this complex multi-system condition. Emerging research evidenced the persistence of symptoms, regardless of severity of initial infection (Arnold et al., 2021; Salamanna et al., 2021). At the time of writing, estimates from the Office of National Statistics suggest that around 2.9% of the UK population (1.9 million people) are experiencing long COVID symptoms (Office for National Statistics, 2023). Furthermore, a review synthesising global evidence found that, on average, around 45% of patients who previously contracted COVID-19 (both hospitalised and non-hospitalised), experienced at least one prolonged symptom (O’Mahoney et al., 2023). In 2020, it was quickly highlighted that primary care services needed better guidance, resources and support to be able to effectively manage the increasing clinical demand of this emerging condition (Ladds et al., 2020). In response to the wide variety of long COVID categorisations and definitions being amassed over the course of the pandemic, in October 2021, the World Health Organisation (WHO) published a working clinical case definition of long COVID (post-COVID condition). The definition was developed through a global consensus process, to optimise the recognition and care of individuals experiencing long COVID. Within this working definition, the list of common symptoms includes fatigue, shortness of breath and palpitations, as well as 22 others (World Health Organization, 2021). Despite significant research advances, many aspects of the condition remain enigmatic, with neurological and neuropsychiatric symptoms often considered to be some of the most complex. Neuropsychiatric symptoms reported in a long COVID meta-analysis included mental health symptoms such as depression and sleep disturbance (Natarajan et al., 2023).

To address knowledge gaps, further funding and research resources have been allocated to better understand this quickly evolving field, and researchers have advocated for the use of a living systematic review (LSR) of long COVID research (Natarajan et al., 2023), which can be continually updated to incorporate new evidence as it is available.

### Exploring parallels with other conditions

In the face of uncertainty, exploring the parallels between long COVID and other better-understood conditions could provide a useful mechanism with which to frame the experience and healthcare needs of patients. For example, parallels have been drawn with patients’ experiences of cancer, and in particular the concept of ‘Survivorship’ (Sounderajah et al., 2020). Harada et al. (2022) observed an opportunity to utilise knowledge gleaned from long-term cancer survivors to inform an understanding of long COVID care. Harada et al. suggested establishing decision-support pathways and early referral to rehabilitation care, similar to those recommended for cancer survivors. Stemming from this, Iqbal et al. (2021) specifically explored the survivorship burden of long COVID, highlighting the importance of public education to reduce stigma.

Through parallels, the identification of theories can reveal deeper layers of the patient experience and their journey through illness. The phenomenology of the illness journey was explored by Moore et al. (2013), who identified three existential modes of being, which describe the lived experiences of cancer patients in a palliative care setting: *Drifting*, *Sheltering* and *Venturing. Drifting* describes patients’ initial entry into hospice and the fear they may never return; the chaos of uncertainty about the course of their illness and their outcomes; and alienation from friends, family and their own body. *Sheltering* describes a sense of comfort found in the certainty of care provided, and a sense of safety found within the physical environment of the hospice. Finally, opportunities to *Venture* provided patients with a chance to see beyond their illness and to begin learning to ‘live’ again, despite the constraints of illness. These three modes reflect how patients perceived and related to their changing world and have been widely cited within cancer and chronic illness literature (Bell et al., 2018; Espeso, 2022; Missel et al., 2022). This characterisation of the illness journey may provide valuable insights when paralleled with the patient journey of those living with long COVID.

Identity transformation experienced by patients with chronic fatigue and fibromyalgia provides additional opportunity to explore parallels, to explore what Asbring (2001) called a ‘disruption in life’. Acknowledging the ‘transformative experience of illness’ (Carel et al., 2016), long COVID can be seen as an illustrative situation in which identity conflicts occur.

### Psychological impact of long COVID

The presence of ongoing psychological symptoms in patients with long COVID has been widely reported. A qualitative analysis of the experience of patients’ living with long COVID who had been referred to psychology services (Skilbeck et al., 2023) identified themes including ‘living with uncertainty’, a ‘loss of identity’ and ‘regaining control’. The impact of limited healthcare information available for patients at the time was also emphasised.

Houben-Wilke et al.’s (2022) observational study of long-term psychological outcomes post COVID-19 conducted in the Netherlands found that a considerable number (35%–47%) of patients (both hospitalised and non-hospitalised) with long COVID had symptoms of anxiety, depression and post-traumatic stress disorder (PTSD) at 3- and 6-month follow-up. While PTSD risk was seen to reduce as time went on, symptoms of anxiety and depression remained high at 6-months follow-up. These results evidence the prolonged psychological impact experienced by long COVID patients.

Additionally, a study exploring the lived experience of ‘brain fog’ following COVID-19 (Callan et al., 2022) found that alongside the impairment of memory, attention or executive function, participants also described associated feelings of guilt, shame and a loss of identity and self-worth. The psychosocial impact on participants was seemingly intensified by the invisibility and fluctuation of symptoms. The majority of these participants had never been hospitalised with COVID-19. It is important to understand what a hospitalised patient cohort might reveal about the overall lived experience of hospitalisation during the pandemic, and how this impacted on their subsequent experience of long COVID.

The influence and interplay of psychological and socio-environmental factors on health and illness is depicted in the biopsychosocial model of health (Engel, 1977). This model acknowledges individual differences and contexts (Wade and Halligan, 2017) and through this lens, a more nuanced and comprehensive understanding of the wider impact of long COVID may be gleaned (Thurner and Stengel, 2023). The adoption of this model of health is recommended for long COVID exploration, to emphasise patient centred research, enable more targeted support and enhance understanding of effective treatment for patients (Skilbeck et al., 2023).

A strong sense of personal identity and self-conceptualisation have been closely linked to psychological wellbeing (Ryff and Keyes, 1995). Theoretical conceptualisations of the ‘self’ could provide insights into the complexities of the psychological impact of long COVID on patients. It has been argued that situations of conflict can become a catalyst for identity crises in which a person questions their definition of self (Baumeister, 1997) and this is borne out in long COVID literature. Previous research using a biopsychosocial approach in the study of long COVID have demonstrated the prevalence of psychological struggles, with many studies highlighting recurring themes of a loss of, or transformation of self-identity (Callan et al., 2022; Houben-Wilke et al., 2022; Kingstone et al., 2020; Spence et al., 2023).

### Ongoing research need

The enigmatic quality of long COVID is intensified by significant variation in patient experience, including symptom longevity and severity, as well as other contextual factors such as access to healthcare (Turk et al., 2024). There is a persisting need for further research to capture this nuance and improve the management and wellbeing of patients with long COVID (Lancet, 2023).

Within the broadening landscape of research in this area, further work is needed to uncover experiences of long COVID across a wider spectrum of timepoints and contexts during the pandemic and beyond. Existing qualitative research on long-COVID largely reflects the views of participants from self-selecting online communities who provided a critically important resource when the need for knowledge was greatest. However, this has resulted in less qualitative evidence concerning the long COVID experiences of those who were initially hospitalised with COVID-19.

There is a need to better understand people’s experience of hospitalisation with COVID-19 as well as support in the community post-discharge, including any unmet physical, psychological and emotional needs they may have. In this project we aimed to explore the lived experience of a cohort of patients who were hospitalised with COVID during the pandemic, and who subsequently developed long COVID symptoms, and their psychological needs.

## Methods

This qualitative study was designed to explore the experiences of hospitalised patients who had persistent COVID-19 symptoms and who had returned to the community. The sample for this study were drawn from the DISCOVER study cohort, a separate observational COVID-19 study that recruited hospitalised patients across two UK hospitals. One of the most important findings from the DISCOVER study at 12-week follow-up clinics was that COVID-19 related symptoms persisted in nearly three-quarters of the study participants (Arnold et al., 2021). Participants were identified from this cohort as having insight into the lived experience of hospitalisation during the pandemic and transitioning back to the community post-discharge. At the time there was little evidence on what that experience was like for patients, and what their support needs were, which provided the rationale for this qualitative study.

A qualitative design was chosen to facilitate a deeper insight into the behaviour and experiences of patients with long COVID through a person-centred approach (Renjith et al., 2021). The importance of patient testimony and validation in long COVID research is strongly emphasised in work by Ireson et al. (2022). Qualitative findings from their analysis of patient experiences of long COVID highlights the need for ‘epistemic humility’ and the continuation of collaborative partnerships with patients in future long COVID research.

This study received ethical approval from London-Chelsea Research Ethics Committee (REC reference: 21/PR/0555). Informed verbal consent was recorded for all participants, detailed further in data collection section.

### Sampling and recruitment

In-depth, semi-structured telephone interviews were conducted with patients who had been hospitalised between April 2020 and February 2021. The sample size was informed by information power (Malterud, 2016), focusing on the relevance and quality of the information, according to the research aims of the project, the specificity of the sample and application of theory. Inclusion criteria were patients aged 18 years and over, who were hospitalised with proven or suspected COVID-19 infection, who had long COVID symptoms as identified during follow-up in the DISCOVER study and who had previously consented to contact about future research. Those excluded were patients who lacked capacity to give informed consent, or who could not converse fluently in English. Participants were selected from the DISCOVER cohort, based on a sampling framework that included age range, gender, ethnicity and existence of long COVID symptoms. Patients were selected to ensure diversity in the sample. AM sent potential participants an information pack (including a cover letter, patient information leaflet and consent form) via email, or to their postal address. Interested participants then contacted a study team member (Andrew Moore-AJM) for further information and to arrange an interview.

### Data collection

Prior to the interview, the interviewer and participant completed a verbal consent process for audio-recording of the interviews and publication of anonymised quotations. Informed verbal consent was audio-recorded, and a paper copy completed at the same time by the researcher. A copy was then sent to the participant for their records. Interviews were conducted by the senior author AJM (male, PhD) who is an academic researcher and methodologist with extensive experience in conducting qualitative research with patients with chronic illness. AJM had no relationship to the participants before this study. Interviews took place via telephone or Microsoft Teams. The topic guides were informed by the scientific literature on COVID-19 and developed in collaboration with clinical team members and members of the study Patient and Public Involvement group (which included three previously hospitalised patient representatives with lived experience of hospitalisation and long COVID symptoms). The topic guide was piloted in the first two interviews. No refinements were necessary (see Appendix A). Topic areas included experience of hospitalisation, long COVID symptoms, experiences of support or support needs, views on vaccination, and concerns about the future and the impact of long COVID.

### Data analysis

Interview audio-recordings were transcribed verbatim, anonymised and imported into QSR NVivo data management software. Transcripts were analysed using thematic analysis (TA).

Through the TA process, authors initially familiarised themselves with the transcripts. Following this, transcripts were coded before being grouped, using inductive methods, into categories (Braun and Clarke, 2006, 2014). We took a collaborative approach to analysis, to facilitate a rich and nuanced reading of the data. AM initially coded the manuscripts, with a portion of them independently coded by AJM. Initial codes were reviewed, and the transcripts were then re-coded. After each interview AJM also kept written field notes to record first impressions, notable events, analytic thoughts or tentative hypotheses. The wider conceptualisation of this project was also considered from a psychological perspective aided during the analytical process by reflection on existing understandings of other chronic conditions, and phenomenological themes. This included the ‘existential modes’ of *drifting*, *sheltering* and *venturing*, that characterised the journey of palliative care patients’ through hospice, from a previous study by Moore et al. (2013). The process of phenomenological reflection informed our interpretation of the participants’ experiences of their hospitalisation and subsequent experiences of long-COVID, and the final realisation of the themes, and sub-themes included here.

### Patient and Public Engagement

Three previously hospitalised patient representatives with lived experience of long COVID symptoms formed a remote public and patient involvement group for this study. Their involvement included reviewing patient facing documents, advising on recruitment and discussing the emerging themes from the interviews.

## Findings

Twelve patients were interviewed between October 2021 and June 2022. Interviews lasted between 21 and 88 minutes (mean average 54 minutes). Adults were interviewed about their experiences of being previously hospitalised with COVID-19 and the impact of their ongoing long COVID symptoms. Participants were aged between 42 and 81 years, and 58% were female. Participant demographics are included below in Table 1, including pseudonyms. The date of participants’ initial hospitalisation with COVID-19 has been included to contextualise their experience of admission within the pandemic timeline.

**Table 1.** Demographics of interview participants.

Pseudonym	Gender	Age group (years)	Date of initial COVID hospitalization
Pauline	Female	74–81	April 2020
Steven	Male	42–49	April 2020
Claire	Female	50–57	April 2020
Alison	Female	58–65	April 2020
Rebecca	Female	50–57	June 2020
Jennifer	Female	74–81	October 2020
Sally	Female	50–57	October 2020
Anna	Female	58–65	November 2020
Darren	Male	58–65	November 2020
Philip	Male	50–57	November 2020
Richard	Male	42–49	November 2020
John	Male	58–65	February 2021

Many commonly acknowledged long COVID symptoms were present in participants’ accounts, including memory and cognitive deficits. Participants describe losing their train of thought, and difficulty with word retrieval were also frequently discussed, as well as reduced tolerance for physical exertion, and fatigue. A symptom less commonly referred to in the existing literature but experienced by some participants was a change in tone of voice. (For full list of symptoms reported by the participants see Appendix B.)

The psychosocial impact of COVID on patients was framed by their experience of hospitalisation and their post-hospitalisation recovery, acknowledging the impact of the patient journey prior to discharge. As a result, the overarching theme developed through this analysis is ‘Existential Crisis’, encapsulating the chaos of uncertainty and seismic shifts in lifeworld perspectives, experienced by hospitalised patients who then developed long COVID.

Within this overarching theme, three interconnecting themes were developed to characterise the elements of existential crisis described by the participants: Facing Psychological Threat, Seeking Legitimisation and Forging a path through uncertainty.

Diagram:


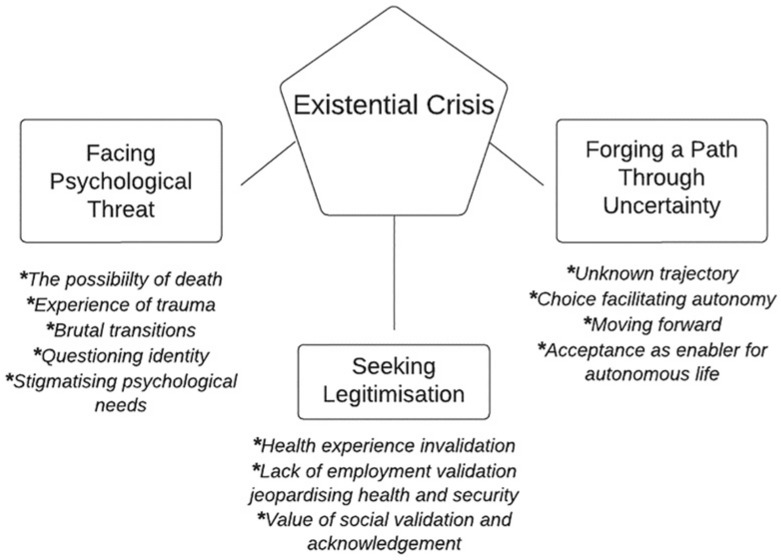


Each theme is described below alongside tables containing illustrative quotations from the interviews. Attributed names are pseudonyms. Quotation Tables 1 to 12 can be found in Supplemental Material.

### Facing psychological threat

When discussing their admission to hospital for COVID and subsequent discharge home, participants described a loss of psychological security throughout their narratives. Participants described a fear of entering the hospital and a pervasive uncertainty about whether they would survive. This is particularly evident in the descriptions of those hospitalised within the first wave of the pandemic, who described their fear that they may not survive. For Claire, life was rendered unrecognisable through her experience of waking up from an induced coma in the ICU:

I remember being woken up and I just remember these incredibly bright lights and people were obviously wearing full PPE but I didn’t quite know what it was because it just looked crazy. People were in these masks and suits (…) I can’t really describe it, but it was just like this alien world. (Claire)

This emphasises Claire’s loss of any sense of familiarity or continuity in her social world. Claire’s uncertainty about the situation and her surroundings is akin to Alison’s expression of bewilderment and disorientation during hospital admission (Supplemental Table 1).

Being confronted with the possibility of dying and being witness to the death of others further added to participants’ fear and uncertainty during admission. Both Alison and Richard described how on the ward they had been surrounded by people who were dying (Supplemental Table 1). They described how the gravity of the situation was visible in the facial expressions of the health care professionals who were caring for them.

Claire, Steven and Alison all acknowledged the possibility that they might die during their hospital admission with COVID-19. Claire, Steven and Alison were all hospitalised early on in the pandemic (April 2020) when very little was understood about COVID-19 and prior to the approval of the use of Dexamethasone as a COVID-19 treatment. It is possible that the timing of their hospitalisation contributed to their concerns.

Patients described how they quickly tried to make sense of a rapidly changing and unfamiliar situation, while struggling to breathe, and to confront the possibility that they might die. Some who were escalated to intensive care were asked whether they wanted to be resuscitated if they went into cardiac arrest – a discussion they were not prepared for and which they found profoundly disturbing. Reflecting on his experience, Darren felt that in a similar situation some less ‘robust’ patients might not have been able to make such a decision with sufficient clarity and suggested that patients should have had an advocate present when being asked about DNARs (Do Not Attempt Resuscitation) (Supplemental Table 1).

Experience of hospitalisation and admittance to the ICU during the pandemic, were described as traumatic and had a profound and lasting psychological impact (Supplemental Table 2).

Richard described being aware of those around him dying, while struggling over a period of 3 weeks to maintain his own breathing, something he described as frightening, and mentally and physically challenging. Following similar experiences Darren described how, following his traumatic experience in hospital, the prospect of going home was also a cause for anxiety. There was a sense from some participants that they were unprepared for discharge and had difficulty coming to terms with the transition (Supplemental Table 3).

Darren described the transition as:

very daunting … very challenging and it wasn’t something that I was prepared for. (Darren)

He goes on to describe how he felt physically and mentally ‘depleted’ by COVID, and that in addition to PTSD, he was anxious about ‘going back into the real world’ (Supplemental Table 3).

Patients in this study also described how their sense of self had changed. This was often invoked by their post-admission reflection during recovery and provoked by the transition back home. For some, their doubts stemmed directly from the impact of ongoing symptoms, including fatigue, breathlessness and brain fog. These reflections suggest a fear that they were fundamentally, and unalterably, changed, both physically and mentally by their experience and long COVID.

Questions of identity arose in patient narratives (Supplemental Table 4), with a strong sense of uncertainty running throughout. Claire’s question, ‘*Will I come back again?*’, suggests a loss of personal identity and a doubt at ever returning to her ‘*old self*’. This sentiment was echoed by other participants. In Rebecca’s description however, there is a sense of certainty and acceptance in the transition, as she talks about dealing with the reality of a new identity as a ‘*different person*’.

While some patients wanted psychological support, there were some who expressed hesitance. Rebecca resisted the suggestion of a need for psychological support, initially perceiving this as delegitimising their physical symptoms (Supplemental Table 5).

The idea that acceptance of psychological support may diminish the validity of claims of physical symptoms, implies a higher societal importance being placed on measurable and concrete markers of illness. It also echoes a perceived stigma towards psychological symptoms, which may be amplified in patients with long COVID due to the uncertainty surrounding the condition. The stigma depicted here arises from the patient themselves.

### Seeking legitimisation

While facing psychological threat, many participants also demonstrated a desire for validation and wider recognition of their long COVID symptoms. The nebulous understandings and definitions of long COVID at the time led to a perceived lack of legitimisation. Existing validation from sources including health professionals, employers and the wider community was suddenly withdrawn, and participants perceived a growing dismissal of their lived experience. This brought up feelings of frustration at the need for continual justification. Implicit within this, was the overriding desire for their lived experience to be acknowledged and understood (Supplemental Table 6).

Participants’ experience of consulting with medical professionals for long COVID, often related to how validated participants felt with their condition. Due to the complex nature of long COVID and the vast array of symptoms, close contact with healthcare professionals provided some with reassurance, acknowledgement and a chance to feel heard. Whereas others experienced frustration with a lack of clarity and responsibility for their care provision.

The uncertainty around the permanency of long COVID symptoms was a concern for those with anxieties about their financial security. Negative perceptions of employers’ response seemed to play a significant role in this uncertainty. Although some felt secure in their employment, others felt misunderstood or were left fighting for acknowledgment, with their health and sense of security in jeopardy (Supplemental Table 7).

There were also positive experiences with employers. Richard describes how he was reassured and told to take time to recover properly:

they were very, very helpful. They kept telling me not to rush back to work which was really good and in the end, I think I had about six to eight weeks off. It was very much about me recovering and concentrating on myself. (Richard)

A lack of external validation from employers or from wider society resulted in many participants relying on the social validation received through the acceptance and support of their family, friends and neighbours. Jennifer discussed the validating impact of receiving ‘check-in’ calls from her church (Supplemental Table 8). The contact provided reassurance, and she placed importance on the motivation behind the behaviour.

However, some sources of social validation and support such as online long COVID support groups proved to be unhelpful as described by Claire. Claire felt her experience of the online support group lowered her mood as conversations focused on members’ struggles with symptoms. She felt most other people in the group had not been admitted to intensive care, and that she was ostracised because of her experience as it did not fit with the common experiences of others in the group. She felt there was an element of ‘competition’ between social comparisons that she experienced as alienating.

### Forging a path through uncertainty

Participants reported being uncertain about their future because of a lack of control over their lives. Some also reported feeling anxious and depressed in response to the unpredictable trajectory and severity of their symptoms (Supplemental Table 9).

Darren talked about the longevity of symptoms and his frustration that more could be done to support people with long-COVID including leveraging peer support from those who have lived experience:

Not everyone is like me and I accept that. The people I met [at long COVID clinic] were from all different social and academic backgrounds but I think that more should be leveraged of the people who have gone through it and want to give back or who have survived it and want to give back (…) It’s like trying to grow a pearl without a piece of grit in an oyster. They’ve got the grit and they’ve got a number of us who have been through it but there has been no thinking that way to think about leveraging that overwhelming desire to give back that those of us who have survived want to do. (Darren)

With uncertainty in so many areas of life with long COVID, vaccine choice (both hesitancy and uptake) offered an opportunity for participants to assert some control through an active and independent decision (Supplemental Table 10).

A desire for the safety of oneself and others, whether through preventative measures or avoidance, echoed across participants’ accounts. Although there were opposing views, decision-making around vaccination provided an opportunity to exercise autonomy in the face of uncertainty. Claire described an increased perception of safety after receiving a vaccination, whereas Pauline’s perception of safety was underpinned by her belief in avoiding vaccination. Fear of further hospitalisation or side effects from the vaccination drove decision making.

Despite some participants feeling despondent after their transition home, and some initial fatigue-management behaviours, there was also a sense that many took practical steps, such as establishing target-focused exercise goals, to reintroduce a sense of control and progress (Supplemental Table 11). This was echoed in participants’ narratives which described moving forward, regaining and rebuilding their lives. Their pursuit of autonomy involved a shift in attitude through acceptance, and acknowledgement.

However for others, an inability to fully self-govern, coupled with the uncertainty of the longevity of symptoms was difficult to manage. Claire struggled with attempting to regain her sense of self and to accept her situation, describing instead an inner ‘resistance’ and ‘continual fight’ against her symptoms (Supplemental Table 12).

Others spoke of accepting their current situation, and symptoms, and getting on with life, while remaining hopeful that things will improve with time. John’s mention of how close he had come to dying, suggests that ‘what [he is] left with’ was easier to cope with.

This analysis sheds further light on the psychological need of previously hospitalised patients, as well as the importance of validation, acceptance and autonomy when managing uncertain long COVID symptoms.

## Discussion

This study set out to explore the lived experience and psychological needs of patients who were previously hospitalised with COVID-19, and subsequently developed long COVID symptoms. Our thematic analysis led to the development of an overarching theme of *Existential Crisis* and subthemes of *Facing Psychological Threat*, *Seeking Legitimisation* and *Forging a path through uncertainty.* These findings highlight the psychological impact and needs of patients’ during and after hospitalisation and the long-COVID experience. Existential threat is depicted through patient testimonies of a prolonged journey of illness, characterised by uncertainty, living through a rapid life-threatening situation and transformation within the self.

### Shedding light on the long COVID journey for hospitalised patients

When orientating the findings of this study alongside Moore et al.’s (2013) modes of existential being (*drifting*, *sheltering* and *venturing*), light is shed on participant journeys through the long COVID experience. Within participants’ journeys, the *drifting* phase is evident through their experiences of fear in entering hospital, the alien context, and their uncertainty about whether they would survive. This sense of drifting often continued following discharge home, as participants experienced a loss or change in their sense of self and trying to cope with symptoms often without the psychological and physical support they wished for. Although a sense of ‘sheltering’ (characterised by a certainty of care, and sense of safety and comfort) was found at times through the support of family and friends, and the provision of vaccines, this was also undermined by participants’ perceived lack of legitimisation, or social validation. Examples of *venturing* were described by participants who had begun to ‘re-enter the world’, accepting their symptoms and the new sense-of-self, with some describing an attitude of hope and determination to ‘move on’ and ‘move forward’. Framing the patient journey around these phases enables gaps in support to be highlighted, demonstrating the importance of reducing participant’s fear, ensuring that patients’ sense of security and safety during hospitalisation is protected, and that post-hospitalisation support includes psychological support. Through this, they can be equipped to advocate for their needs more effectively, throughout their recovery.

### Situating our findings within existing literature

Asbring (2001) depicts illness as a ‘disruption in life’, while Carel et al. (2016) suggests this disruption is also a ‘transformative’ experience. The experiences that patients described in this study similarly suggest that living with long COVID post-hospitalisation is also transformative in the sense that they experience a shifting identity and altered sense of self.

Additionally, our findings of the presence of psychological threat and extreme uncertainty in people with long COVID mirror the experiences of participants within other studies on COVID-19 (Callan et al., 2022; Skilbeck et al., 2023; Spence et al., 2023). As well as supporting these existing findings, the PELCO study builds on previous evidence by adding insights from the lived experience of those who were hospitalised prior to their developing long-COVID. We suggest that their experience of hospitalisation, characterised by existential crisis, may have compounded, and set the psychological tone for patients’ subsequent response to the development of long-COVID symptoms, and the need for better psychological support.

The theme *Seeking Legitimisation* supports findings in the literature of the importance of recognition and validation of long COVID symptoms (Iqbal et al., 2021) Previous studies have also found that patients with long COVID experience a lack of validation (Harada et al., 2022; Ireson et al., 2022) and we support the notion that clinicians must acknowledge patient concerns and symptoms, and validate their need for support.

In contrast to findings from other research in this area with non-hospitalised communities, participants in this study seemed to gain less comfort and validation from online support groups. This suggests a potential disparity in the support needs of those who were hospitalised, however there is extensive evidence (Houben-Wilke et al., 2022; Iqbal et al., 2021; Ireson et al., 2022; Skilbeck et al., 2023; Spence et al., 2023; Thurner and Stengel, 2023) to argue that many long COVID patients experience a psychological struggle, including both hospitalised and non-hospitalised populations.

This paper’s overarching theme of *Existential Crisis* resonates with the ‘survivorship’ conceptualisation of the experience of long COVID (Harada et al., 2022; Sounderajah et al., 2020). Inherent in this is the validation of the lasting impact of the initial COVID-19 infection, while also encapsulating the wide-ranging effect of prolonged symptoms. In our study, participants’ expressions of trauma and psychological struggle were linked, in part, to their experience of hospital admission. This appeared to be emphasised in patients who were hospitalised earlier in the pandemic and those admitted to ICU. This strongly supports calls for earlier psychological intervention for patients with long COVID (Houben-Wilke et al., 2022).

### Strengths and limitations

Our qualitative approach enabled us to explore the complex lived experiences and views of the participants with a focus on the psychological impact of hospitalisation and long COVID. The study benefitted from expertise from a multidisciplinary team including the perspectives of researchers with a background in psychology (AM), sociology of health and chronic illness research (AJM), and a respiratory clinical academic with experience in leading an observational COVID-19 cohort study (DTA).The use of Moore et al.’s Existential Modes of Being also facilitated and deepened the interpretation of the data and more broadly adds to the literature on patient journeys through chronic illness.

Our sample of 12 participants was sufficient to achieve information power, based on the specific aims of the study, the density and richness of the data. There are inherent diversity issues and sample limitations with qualitative studies in this area, with female participants representing between 70% and 91% (Callan et al., 2022; Ladds et al., 2020; Skilbeck et al., 2023; Spence et al., 2023; Thomas et al., 2023). This homogeneity in reporting is reflective of the comparative association of higher incidence of long COVID in women (Bai et al., 2022). We achieved a closer balance within our sample (58% female), drawing from two geographically diverse hospitals in the Midlands and Southwest of England. However, it is important for future qualitative research to reflect the full spectrum of long COVID patient experience including populations with diversity in gender, ethnicity, geographic placement and socioeconomic status.

Some recruitment difficulties were experienced, potentially related, in part, to overburdening of research participants who had also been approached to participate in other COVID-19 research. Due to the sample size, we were unable to draw out differences in subsequent long COVID experiences between those hospitalised earlier in the pandemic compared to those hospitalised slightly later as their individual experiences vary. Recruitment burden is something to continue to be mindful of in future qualitative studies and future pandemic events. However, the authors have confidence in the information power of the achieved sample, due to the rich data gathered.

### Conclusions and future directions

The findings of this study add to the growing body of evidence on the significant psychological burden of living with long COVID. The qualitative methodology was integral to eliciting these findings. Our study emphasises the level of psychological impact amongst this patient group. These findings highlight the need for early psychological review and intervention to ease the ‘brutal transition’ of discharge experienced by many of the patients in this study. This supports similar findings (Houben-Wilke et al., 2022) that suggest that early psychological assessment and intervention should be available for patients following pandemic events such as COVID-19.

We recommend that wider support is established and accessible in the community to provide patients with targeted management strategies for physical long COVID symptoms. Providing consistent implementation of multi-disciplinary support could validate patients’ post-discharge health needs and empower them to advocate for their care more readily. However, the authors acknowledge the extensive resourcing implications for the large volume of patients this would include, as well as the barriers to implementation of this.

Our findings also suggest the importance of further planning for future pandemics to ensure the presence of patient advocates during hospitalisation at points of critical decision-making such as DNAR (Do Not Attempt Resuscitation) orders. In the likelihood of a future health crisis, further research should explore the most effective psychological support strategies and intervention timelines for patients with consideration to contextual factors including emergency hospitalisation. We reiterate the importance of empowering long COVID patients by validating their lived experience through in-depth qualitative enquiry.

## Supplemental Material

sj-docx-1-hpq-10.1177_13591053241272233 – Supplemental material for Understanding post-hospitalised patients’ experiences of long-COVID – the PELCO study Supplemental material, sj-docx-1-hpq-10.1177_13591053241272233 for Understanding post-hospitalised patients’ experiences of long-COVID – the PELCO study by Alice Milne, David Arnold and Andrew Moore in Journal of Health Psychology

sj-docx-2-hpq-10.1177_13591053241272233 – Supplemental material for Understanding post-hospitalised patients’ experiences of long-COVID – the PELCO study Supplemental material, sj-docx-2-hpq-10.1177_13591053241272233 for Understanding post-hospitalised patients’ experiences of long-COVID – the PELCO study by Alice Milne, David Arnold and Andrew Moore in Journal of Health Psychology
