# Supplementary material for: Understanding post-hospitalised patients’ experiences of long COVID – the PELCO study
Source: J Health Psychol. 2024 Aug 22;30(4):780–93. doi: 10.1177/13591053241272233 (PMC11927023; doi:10.1177/13591053241272233)
Supplement: sj-docx-1-hpq-10.1177_13591053241272233 – Supplemental material for Understanding post-hospitalised patients’ experiences of long-COVID – the PELCO study [file sj-docx-1-hpq-10.1177_13591053241272233.docx]

Understanding **P**ost-hospitalised Patients’ **E**xperiences of **L**ong-**CO**VID -
The **PELCO** Study

Milne, A., Arnold, D,T., Moore, A,J.

**PELCO Study
Appendix A:**  **Topic Guide**

**PELCO Topic Guide - Patients**

**Introduction**: Discuss how the interview will be recorded, issues of confidentiality, anonymisation.

**Aim of the study**: The aim of this study is to understand and characterize the impact of COVID and long term symptoms on people’s quality of life and wellbeing.

**Consent procedure**: Check and audio-record verbal consent to the study.

**Participant information:** Age, employment, ethnic background, onset of COVID symptoms, length of hospitalisation.

1. Can you tell me about your experience of COVID and when you first started to have symptoms?
2. What was your experience of hospitalisation like?
3. What has your recovery journey been like so far? (If struggling, one or two words to describe it?)
4. Can you tell me when you first realised you had Long-COVID symptoms?
5. Can you describe the symptoms for me?
6. Do they change or fluctuate? How?
7. What in your experience exacerbates or provokes the recurrence of your symptoms of Long COVID?
8. Can you describe any support you’ve had from health care or otherwise during and after your hospitalisation? (did you have any support from your GP, physiotherapy?)
9. Have you sought support from any community or online COVID groups?
10. What support do you feel that you need (then and now)?
11. Have you ever attended a Long-COVID support clinic? (different to the DISCOVER follow-up clinics)
12. Have you had the COVID Vaccine?
13. What are your thoughts on having the vaccine?
14. What are your thoughts on the booster vaccine?
15. What are your thoughts about your own level of immunity (*resistance to colds, flu, or other COVID strains*) going forward?
16. What are your concerns for the future for you and your family?
17. How have you coped with the uncertainty that seems to be characterising this pandemic?
18. How do you think the effects of Long-COVID have affected your family or those in your household?
19. What have you done that has helped you, in terms of managing your symptoms?
20. How do you feel about taking part in research during the COVID pandemic?
21. What are the important questions that you feel still need to be answered?
22. What information do you think people need about Long-COVID?

**Conclusion**

1. Is there anything else you would like to add, or anything you wish to talk about that we haven’t covered already?
2. Would you like us to send you a summary of the results of the study once it’s finished?
3. Thank you for participating…END.
4. REAFFIRM CONSENT

**PELCO Study

Appendix B:**  **List of Symptoms Described by Participants**

| Symptoms Described by Participants in Interviews |
| --- |
| Brain fog and memory issues |
| Breathlessness |
| Feeling ‘carbonated’ |
| Fatigue |
| Chest discomfort – tightness, feeling heavy |
| Feeling of ‘cracking at the base of the lungs’ |
| Exacerbation of pre-existing conditions |
| Headaches |
| Joint and muscle pain |
| General mobility reduction |
| ‘Nerve sparking’ |
| Palpitations |
| Reduced exercise tolerance |
| Sleep disturbance and difficulties |
| Voice Alteration |
