# Supplementary figures and images for: Understanding post-hospitalised patients’ experiences of long COVID – the PELCO study
Source: J Health Psychol. 2024 Aug 22;30(4):780–93. doi: 10.1177/13591053241272233 (PMC11927023; doi:10.1177/13591053241272233)

Table 1:

Table 2:

Table 3:

Table 4:

Table 5:


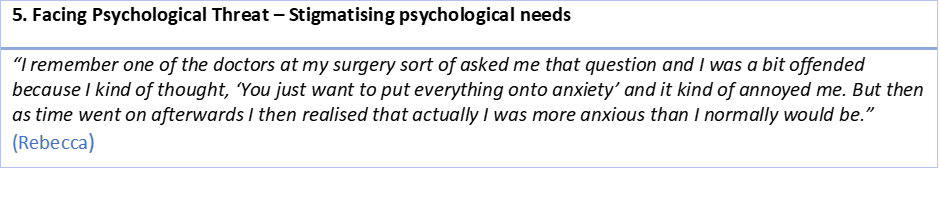


Table 6:

Table 7:


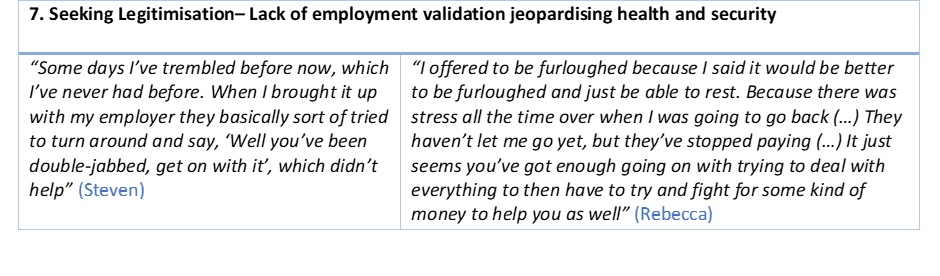


Table 8:


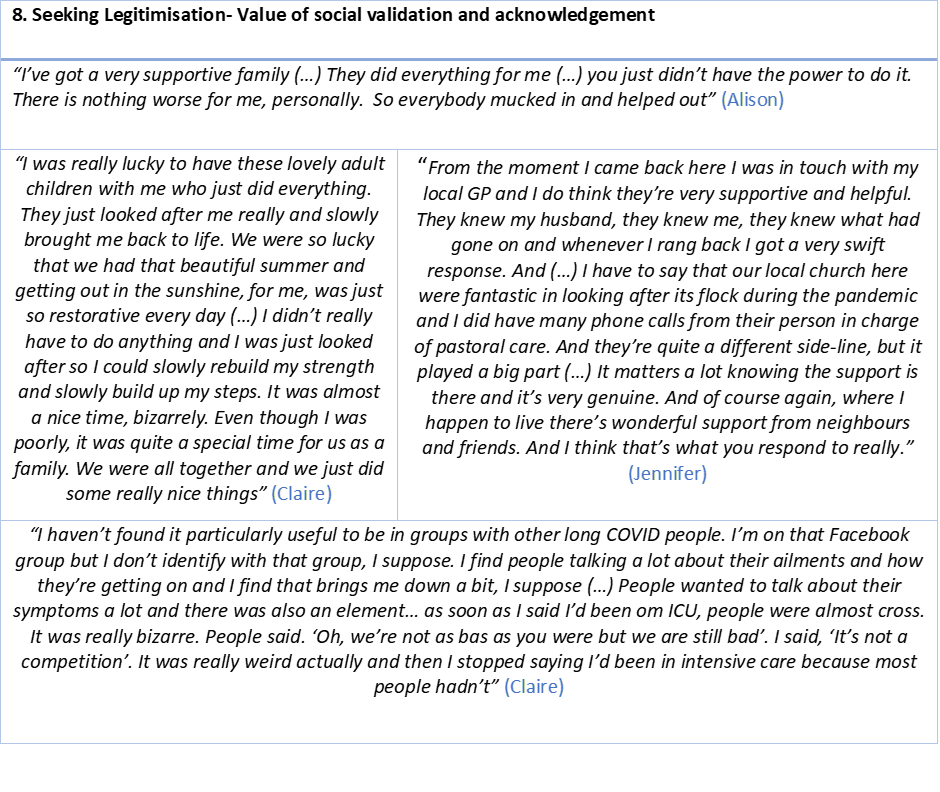


Table 9:


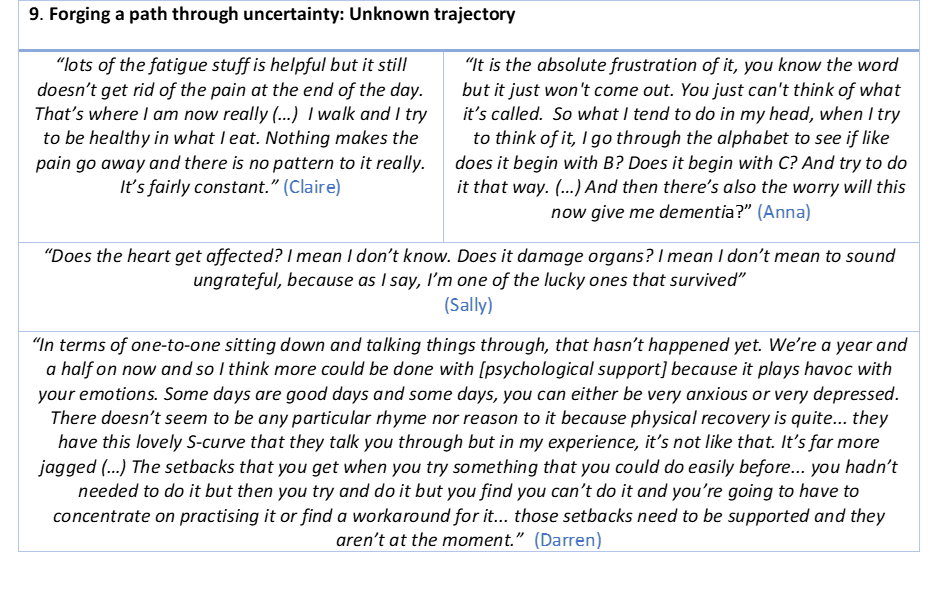


Table 10:


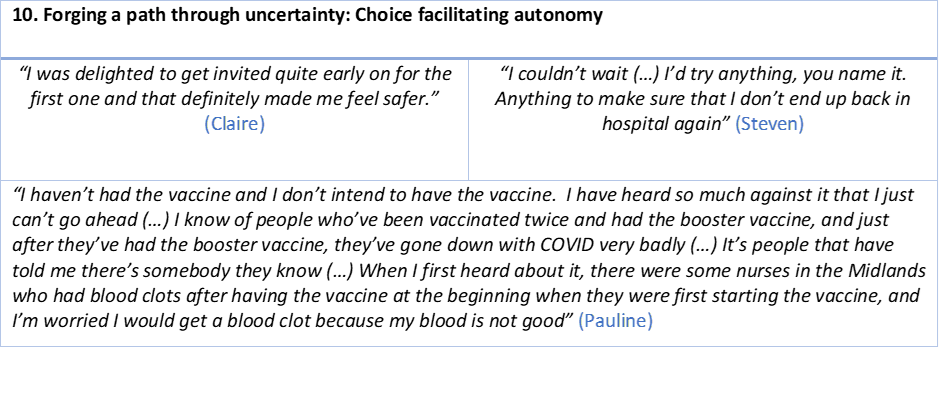


Table 11:


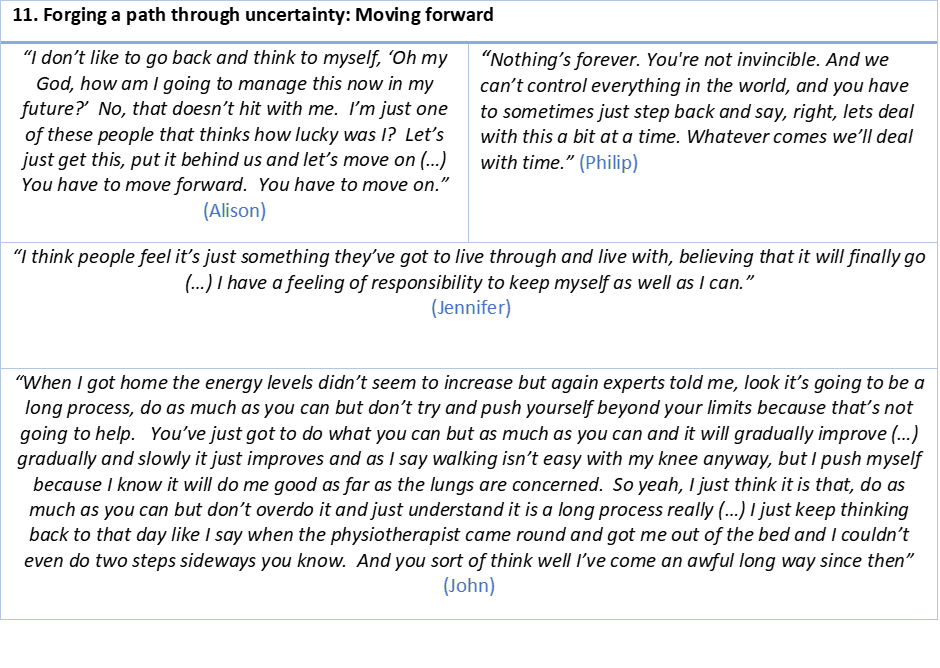


Table 12:


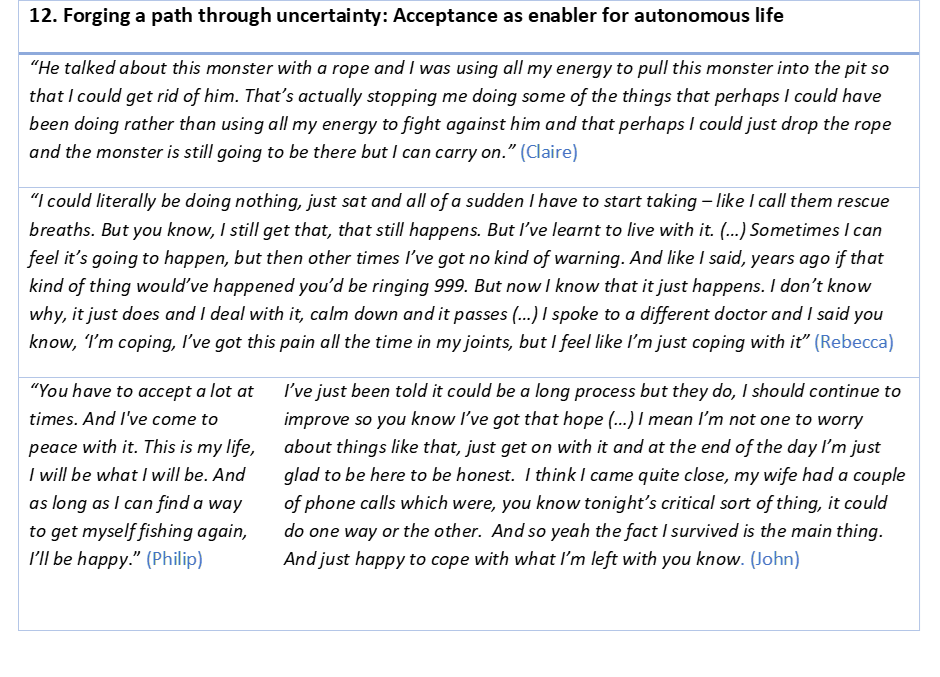

Supplement: sj-docx-2-hpq-10.1177_13591053241272233 – Supplemental material for Understanding post-hospitalised patients’ experiences of long-COVID – the PELCO study [file sj-docx-2-hpq-10.1177_13591053241272233.docx]
